# Dysosteosclerosis Presents as an “Osteoclast-Poor” Form of Osteopetrosis: Comprehensive Investigation of a 3-Year-Old Girl and Literature Review

**DOI:** 10.1002/jbmr.131

**Published:** 2010-05-17

**Authors:** Michael P Whyte, Deborah Wenkert, William H McAlister, Deborah V Novack, Angie R Nenninger, Xiafang Zhang, Margaret Huskey, Steven Mumm

**Affiliations:** 1 Center for Metabolic Bone Disease and Molecular Research, Shriners Hospital for Children St Louis, MO, USA; 2 Division of Bone and Mineral Diseases, Washington University School of Medicine at Barnes-Jewish Hospital St Louis, MO, USA; 3 Department of Pediatric Radiology, Mallinckrodt Institute of Radiology, St Louis Children's Hospital at Washington University School of Medicine St Louis, MO, USA; 4 Department of Pathology, Washington University School of Medicine at Barnes-Jewish Hospital St Louis, MO, USA

**Keywords:** bone remodeling, erlenmeyer flask deformity, osteoclast, osteosclerosis, RANKL, skeletal dysplasia

## Abstract

Dysosteosclerosis (DSS), an extremely rare dense bone disease, features short stature and fractures and sometimes optic atrophy, cranial nerve palsy, developmental delay, and failure of tooth eruption in infancy or early childhood consistent with osteopetrosis (OPT). Bone histology during childhood shows unresorbed primary spongiosa from deficient osteoclast action. Additionally, there is remarkable progressive flattening of all vertebrae and, by adolescence, paradoxical metaphyseal osteopenia with thin cortical bone. Reports of consanguinity indicate autosomal recessive inheritance, yet more affected males than females suggest X-linked recessive inheritance. We investigated a nonconsanguineous girl with DSS. Osteosclerosis was discovered at age 7 months. Our studies, spanning ages 11 to 44 months, showed weight at approximately 50th percentile, and length diminishing from approximately 30th percentile to –2.3 SD. Head circumference was +4 SD. The patient had frontal bossing, blue sclera, normal teeth, genu valgum, and unremarkable joints. Radiographs showed orbital and facial sclerosis, basilar thickening, bone-in-bone appearance of the pelvis, sclerotic long bone ends, and fractures of ribs and extremities. Progressive metaphyseal widening occurred as vertebrae changed from ovoid to flattened and became beaked anteriorly. A hemogram was normal. Consistent with OPT, serum parathyroid hormone (PTH) concentrations reflected dietary calcium levels. Serum bone alkaline phosphatase, osteocalcin, and TRACP-5b were subnormal. The iliac crest contained excessive primary spongiosa and no osteoclasts. No mutations were identified in the splice sites or exons for the genes encoding chloride channel 7, T-cell immune regulator 1, OPT-associated transmembrane protein 1, and monocyte colony-stimulating factor (M-CSF) and its receptor C-FMS, ANKH, OPG, RANK, and RANKL. Genomic copy-number microarray was unrevealing. Hence, DSS is a distinctive OPT of unknown etiology featuring osteoclast deficiency during early childhood. How osteopenia follows is an enigma of human skeletal pathobiology. © 2010 American Society for Bone and Mineral Research.

## Introduction

Dysosteosclerosis (DSS; OMIM % 224300)(1) refers to an extremely rare disorder characterized in 1968 by Spranger and colleagues(2) that presents in infancy or early childhood and features diffuse osteosclerosis with expanded ends of the tubular bones and platyspondyly. In keeping with a type of osteopetrosis (OPT),(3) bone histology during childhood shows unresorbed primary spongiosa and therefore deficient osteoclast action.(4,5) The skeleton is dense but brittle. Patients develop short stature and fractures and can suffer optic atrophy and sometimes cranial nerve palsy, developmental delay, and failure of tooth eruption,(1,6–9) in keeping with “marble bone disease.”(3) MRI has suggested delayed myelination in the brain,(10) seizures have occurred,(11) and various skin lesions have been reported.(11–14) Consanguinity(2,6,12,13,15) and a greater number of affected males (including in two generations) than females(11) suggest that DSS can be either an autosomal recessive trait(1,15) or an X-linked recessive trait,(11) but the genetic defect(s) is not known.(1)

The clinical course and prognosis of DSS are not well understood. In 1978, Houston and colleagues(6) reported a 15-year-old boy whose iliac crest histology at 20 months of age revealed unresorbed primary spongiosa consistent with OPT. Subsequently, however, metaphyseal widening and sclerosis of early childhood changed to greater metaphyseal expansion (extending into the diaphysis) with remarkable osteopenia and cortical thinning by adolescence. In 2008, his follow-up at age 44 years was reported by Lemire and Wiebe,(16) who documented persistence of the widened proximal and distal ends of the long tubular bones with central diaphyses that were not expanded and showed cortical thickening.

Here, we describe our clinical, biochemical, radiologic, histopathologic, and genetic investigation of a young girl with DSS and review the pertinent literature.

## Materials and Methods

Informed written consent for all studies was obtained as approved by the Human Research Protection Office, Washington University School of Medicine (St Louis, MO, USA).

### Case Report

This 44-month-old American girl was admitted to our research center at ages 11, 23, 25, and 35 months. Additional outpatient serum and urine specimens were obtained at ages 27, 30, and 44 months.

### Medical history

The patient was born vaginally at 38 weeks' gestation to a 37-year-old G_3_, P_3_, A_0_ woman. Birth weight was low for gestational age at 5 pounds, 14 ounces, but length 19½ inches.

At 5 months of age, computed tomography (CT) for rapidly increasing head circumference (25th percentile at birth, 97th percentile at age 5 months, despite growth at 50th percentile for both length and weight) reportedly showed prominent ventricles, sulci, and subarachnoid spaces but no space-occupying lesion or hemorrhage.

At 7 months of age, a radiograph (see below) for posttraumatic arm pain prompted a skeletal survey showing metaphyseal sclerosis that was attributed to “rickets.” Vitamin D was prescribed but not taken.

From birth to 8 months of age, the patient had recurrent ear and skin infections and abdominal discomfort believed from colic.

Between 8 and 11 months of age, several episodes of joint-centered limb pain were thought to be from dislocations. On referral to us at age 11 months, psychomotor development was on time. Tooth eruption was not delayed.

At age 20 months, the patient fractured her distal left femur during a fall. Healing was delayed in a full splint.

Ophthalmologic exam was normal at age 28 months. Audiometry had not been performed, but she seemed to hear well, had clear speech, and was bilingual.

At age 30 months, she fractured through dense metaphyseal bone in her right radius and ulna during a fall. Reportedly, healing was slow (casted for 3 months). Parental recollection of further breaks was negative, but radiographs showed previous fractures. Morning stiffness in her lower limbs lasted 2 to 3 hours each day. She had chronic nasal “stuffiness.” Several episodes of erythematous papules involved her lower abdomen and were always on at least one area of her body but usually her belly, legs, or arms. Episodes lasted 3 to 4 days, were not associated with fever, and did not hurt or itch.

At 36 months, she fell and fractured her distal right femur, where dense metaphyseal and more normal-appearing diaphyseal bone joined. Healing was delayed in a long-leg cast for 3 months.

She received acetaminophen for nighttime fussiness but no multivitamins or supplements. She drank filtered municipal tap water. There were no factories near her home, but lead paint had been removed from a nearby house.

At age 44 months (3⅔ years), interval history revealed a fractured left femur from a fall onto a carpeted floor with delayed healing while casted. Subsequently, however, the patient could run. There was generalized morning stiffness and painful ribs.

Her healthy brothers were 6 and 8 years old. The family history was negative for skeletal disease or parental consanguinity. The father was of Turkish descent. The mother's family emigrated to Turkey from Greece 70 years previously.

### Physical findings

**Fig. 1:**
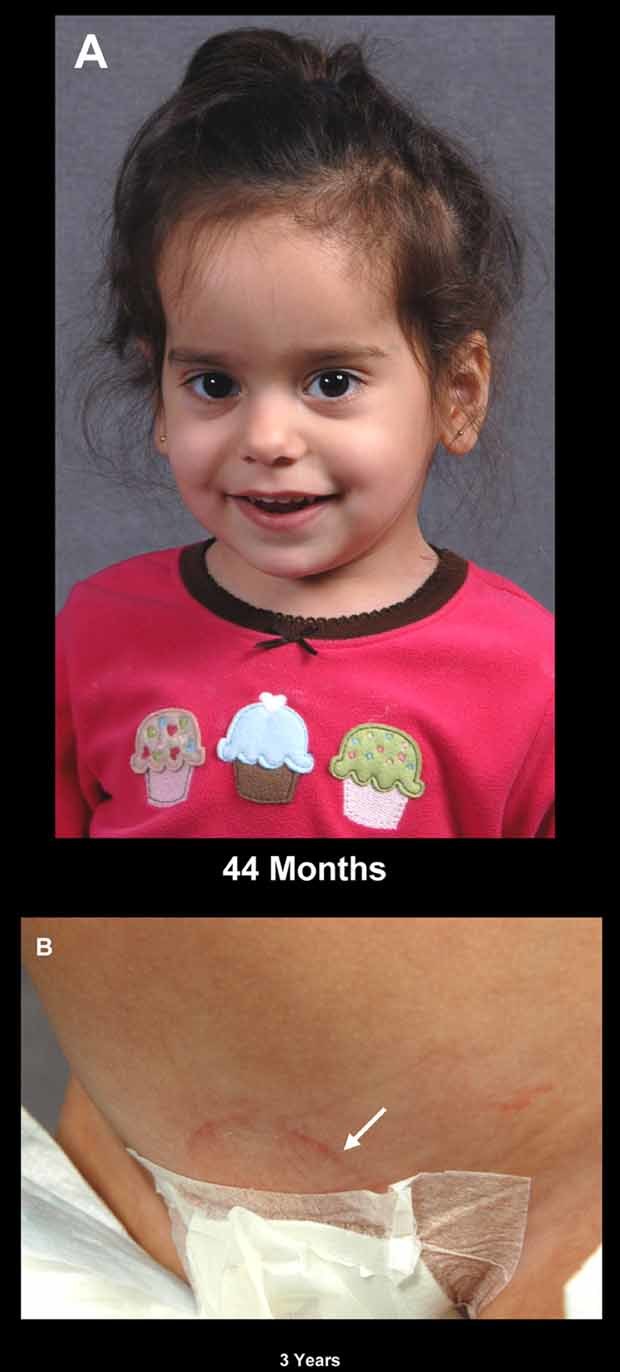
Patient. (*A*) At age 44 months, there is frontal bossing, a small midface, low-set ears, and blue sclerae. (*B*) At age 3 years, the recurring dermatosis (*arrow*) is present on the lower abdomen.

When examined for diagnosis at age 11 months, the patient had a high forehead, frontal bossing, blue sclerae, a flattened nasal bridge, downturned mouth (Fig. 1*A*), a narrow and high-arched palate, and a wide palatine ridge with nine normal-appearing teeth, indicating no delay in tooth eruption. Joint range of motion was full. A dermatosis involved her lower abdomen (Fig. 1*B*).

At age 22 months, she had no palpable cranial sutures, bony tenderness, or genu valgum. Although 50th percentile for weight and length at age 5 months, between 11 and 35 months of age she maintained her weight at approximately 30th percentile of normal, but length decreased from approximately 30th percentile to below the growth curve (*Z*-score = –2.5). The upper/lower body segment *Z*-score increased to greater than 2.0. Arm span was less than 5th percentile. Head circumference *Z*-score decreased from +4.3 to +3.6. Genu valgum was present radiographically as early as 22 months of age.

At age 44 months, weight was approximately 59th percentile, and length was –2.3 SD. Arm span versus height had progressed initially along the 50th percentile, but arm span measurements now showed that her arms were disproportionately short.(23)

### Biochemical testing

Fasting blood was used for all in-patient studies. Biochemical profiles were obtained from our Dade Behring Dimension Xpand instrument (Siemens Health Care Diagnostics, Inc., Los Angeles, CA, USA) using the reference ranges provided by the manufacturer. However, for all key findings, results were contrasted with values from fasting sera obtained in 2006 and 2007 from 34 healthy children and 24 healthy adults.(17)

At age 11 months, the patient was given a diet containing approximately 400 mg of calcium per day to match her home intake estimated from a 7-day food record [Recommended Daily Allowance (RDA) for age = 270 mg]. At age 23 months, intake was approximately 950 mg. At age 35 months, we reduced her calcium intake to 775 mg daily (RDA of 500 mg recommended) to prevent suppressed serum parathyroid hormone (PTH) levels (see “Results”).

To explore whether DSS is an OPT, we assayed serum levels of the brain isoenzyme of creatine kinase, BB-CK (Kit K20, Sebia, Norcorss, GA, USA),(18) tartrate-resistant acid phosphatase (TRACP-5b, Kit 8033, Quidel, Los Angeles, CA, USA),(17) and lactate dehydrogenase (LDH) and aspartate aminotransferase (AST).(17)

Skeletal remodeling was assessed by quantitating serum bone-specific alkaline phosphatase (BAP) using ELISA (Quidel Corp., San Diego, CA, USA) and osteocalcin (Kit LKON1, Siemens Health Care Diagnostics, Inc.) and urine free deoxypyridinoline (DPD, Immulite 1000 Pyrilinks, D Kit, Siemens Medical Solutions Diagnostics, Ltd., Lianberis, Gwynedd, UK).

### Radiologic studies

All radiologic studies, including images obtained elsewhere, were reviewed to assess the nature and evolution of the patient's skeletal disease, including fractures and their rate of healing. Dual-energy X-ray absorptiometry (DXA) of the patient and her parents involved a Hologic QDR 4500-A instrument (Hologic Corp., Waltham, MA, USA).

### Histopathologic studies

At 2 years of age, two 3-day oral courses of demeclocycline separated by 10 days were ingested. Two days after the last dose, a transapophyseal iliac crest biopsy was performed using a Trap-Lok needle (Medical Device Technologies; Gainesville, FL, USA). The specimen was fixed in 70% ethanol and 30% water, embedded without decalcification in methyl methacrylate, and cut into 5-µm sections for staining via the hematoxylin and eosin (H&E), toluidine blue, and von Kossa methods. Unstained 10-µm sections were used for fluorescence microscopy.

### Mutation analyses

Three genes (exons and mRNA splice sites) associated with OPT [chloride channel 7 (*CLCN7*), T-cell immune regulator 1 (*TCIRG1*), and OPT-associated transmembrane protein 1 (*OSTM1*)] had been studied at Connective Tissue Gene Tests (Allentown, PA, USA). We purified genomic DNA from blood leukocytes using the Gentra Puregene DNA extraction kit (Invitrogen, Carlsbad, CA, USA) and examined all coding exons and adjacent mRNA splice sites of *TNFRSF11A* encoding RANK, *TNFRSF11B* encoding OPG, and *TNFSF11* encoding RANK ligand (RANKL) by PCR and sequencing in both directions using published methods and primers.(19–21) Because few osteoclasts were noted on our patient's iliac crest biopsy, the CSF1 and CSF1R genes encoding monocyte colony-stimulating factor (M-CSF) and its receptor C-FMS, respectively, were examined using primers and conditions that we developed (available on request). Furthermore, her bone modeling disturbances and the literature report of resolution of long bone osteosclerosis in DSS(6,16) led us to study the ANKH gene deactivated in craniometaphyseal dysplasia.(22) DNA sequences were evaluated using AlignX software (Vector NTI, Invitrogen) and by inspecting electropherograms.

Genomic DNA copy-number microarray analysis of the proband and parents was performed using the Affymetrix SNP 6.0 chip at the Laboratory for Clinical Genomics, Washington University School of Medicine (St Louis, MO, USA) and the Partek Genomics Suite (Partek, St Louis, MO, USA).

### Literature review

We assessed all previous reports of DSS using original publications obtained from the Becker Library of Medicine, Washington University School of Medicine (St Louis, MO, USA), especially the radiographic reproductions.

## Results

### Biochemical testing

Serum ionized calcium was highest, 5.3 mg/dL (normal 4.7 to 5.3 mg/dL), during the first admission at age 11 months (Table 1), when her estimated dietary intake of calcium significantly exceeded the RDA. With less dietary calcium, serum generally contained lower total and ionized calcium concentrations, but PTH levels fluctuated considerably over ages 11 to 44 months, ranging from 7 to 120 pg/mL (normal 4 to 52 pg/mL; Table 1). Calcium in timed urine collections ranged from 196 to 241 mg/g of creatinine (Cr).

**Table 1:** Longitudinal Biochemical Findings

	Serum							
	
	Total Ca	Ca^2+^	PTH	P_*i*_	ALP	BAP	Osteocalcin	TRACP-5b
	
Age (mos.)	(normal 8.9–10.2 mg/dL)	(normal 4.7–5.3 mg/dL)	(normal 4–52 pg/mL)	(normal 3.4–5.9 mg/dL)	(normal 157–477 IU/L)	(normal 88–247 U/L)	(normal 37–119 ng/mL)	(normal 13–23 U/L)
11	10.1	5.3	34	4.7	213	—	9	8
23	10.2	5.2	33	5.0	162	—	2	—
25	9.1	4.9	120	4.5	210	85	12	2
27*	10.1	—	7	5.2	125	—	—	—
30*	10.0	—	38	4.6	140	—	—	—
35	9.9	5.0	62	4.2	136	41	12	3
44*	9.8	5.0	22	4.4	176	63	6	3

— = not done.

* = non-fasting outpatient serum.

Serum 25-hydroxyvitamin D [25(OH)D] was 28 ng/mL (normal 32 to 100 ng/mL) (Lab Corp., Burlington, NC, USA), with normal levels of magnesium and inorganic phosphate.

Serum alkaline phosphatase (ALP) decreased to as low as 125 IU/L (normal 157 to 477 IU/L) and was intermittently subnormal for age, especially in view of recurrent fractures. Serum BAP, osteocalcin, and TRACP-5b levels all were consistently low (Table 1). Urine free DPD was 18 nmol/mmol of Cr (23 months) and 14 nmol/mmol of Cr (44 months) (normal 7 to 34 nmol/mmol).

Serum BB-CK, LDH, and AST levels were unremarkable, unlike in some OPTs, where elevated values can occur.(17,18) Urine did not contain heavy metals. Serum was negative for lead (Legacy Laboratory Services, Portland, OR, USA).

At ages 23, 35, and 44 months, complete blood counts were normal, including monocytes. Erythrocyte sedimentation rate seemed elevated for a child at 18, 15, and 24 mm/h, respectively (normal 0 to 20 mm/h for young adults), and serum C-reactive protein was consistently normal. Serum contained elevated IgG of 1416 mg/dL (normal 453 to 916 mg/dL) and slightly high IgA of 111 mg/dL (normal 20 to 100 mg/dL) (Lab Corp.).

### Radiologic findings

Radiographic skeletal surveys were available from ages 7, 11, 22, and 33 months. Right hand and wrist films were available at 30 months, left knee at 42 months, and right knee at 47 months.

**Fig. 2:**
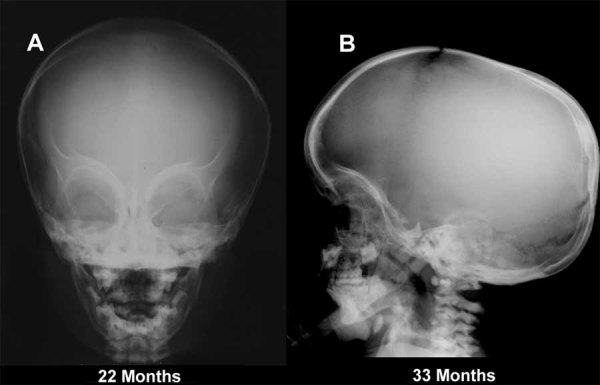
Skull. (*A*) At age 22 months, anteroposterior view of the skull shows sclerosis that affects the periorbital region and skull base. The sinuses are opacified. (*B*) At age 33 months, the neurocranium appears large on lateral view, with diffuse sclerosis greatest at the base. The midface is small, and the mandibular angle is obtuse.

A generalized skeletal disorder was revealed, where every imaged bone was abnormal (Figs. 2 through 5). Prominent findings included basilar sclerosis of the skull and modeling abnormalities (expansion) in the major long bones resembling OPT but also ovoid vertebral bodies characteristic of DSS. The sclerosis must have occurred postnatally because relatively normal iliac bones had formed in utero that subsequently became sclerotic peripherally. The changes per anatomic site are described below.

**Fig. 3:**
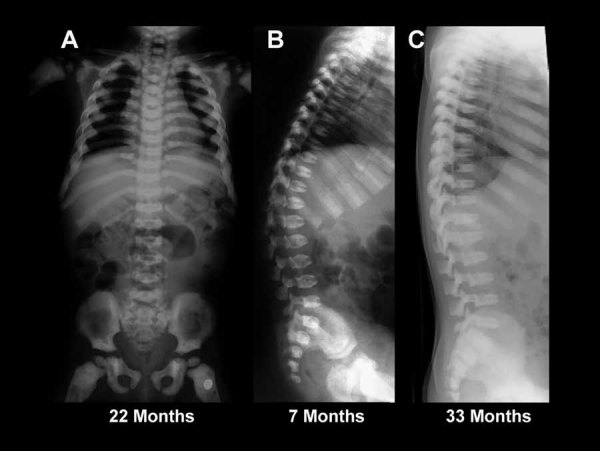
Spine. This anteroposterior projection (*A*) at 22 months shows sclerotic vertebrae, dense ribs with healing fractures, sclerotic medial clavicles, and bone-in-bone appearance in the ilium as seen in some forms of OPT. Between 7 (*B*) and 33 (*C*) months of age, the lateral spine shows sclerosis and flattening of vertebral bodies that increased with age. The vertebral bodies have a pointed configuration anteriorly (greatest at 7 months). Sclerosis affects the entire vertebra.

**Fig. 4:**
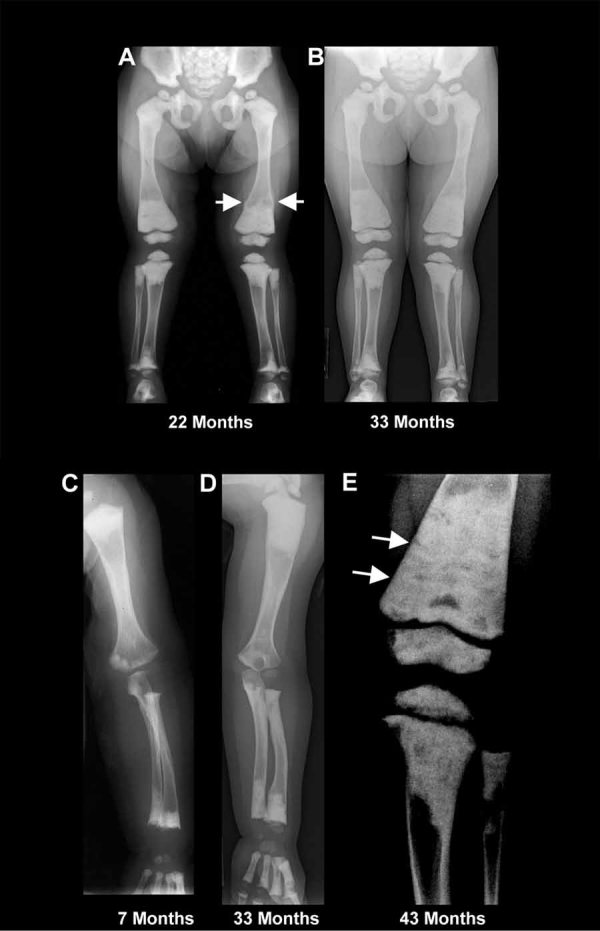
Appendicular skeleton. At 22 (*A*) and 33 (*B*) months of age the lower extremities, and at 7 (*C*) and 33 (*D*) months of age the left upper extremity, show sclerosis greatest at the proximal and distal ends of the bones and metaphyseal expansion from tubulation failure greatest in the distal femurs. (*E*) There are transverse metaphyseal bands of osteosclerosis, as seen in some forms of OPT (*arrows*). A healing fracture featuring focal bone expansion and periosteal new bone formation is present in the distal left femur at the junction of the sclerotic and nonsclerotic bone (*arrows*) (*A*). There is a slight irregularity of the physeal plates and adjacent provisional zones of calcification (*C*, *D*, *E*). Sclerosis in the upper extremities has increased at the ends of the bones between 7 (*C*) and 33 (*D*) months of age. In the leg bones and distal right femur, the junction between the sclerotic and nonsclerotic bone is sharp, but not well defined in the proximal femurs or distal left femur. The junction between the sclerotic and nonsclerotic bone can have a transverse or oblique shape.

**Fig. 5:**
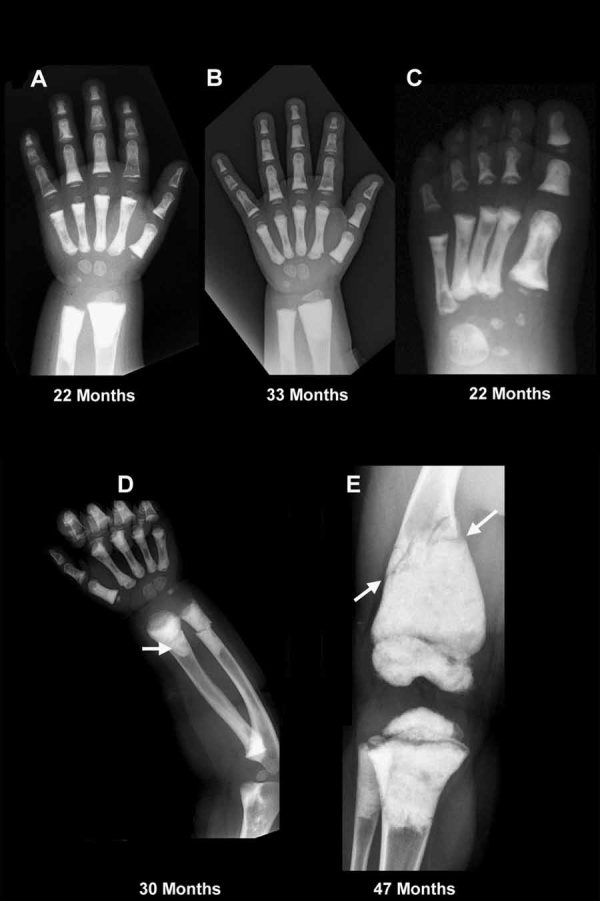
Hand and foot radiographs. At 22 (*A*) and 33 (*B*) months of age the left hand and at 22 (*C*) months of age the left foot show diffuse sclerosis greatest in the metaphyses of the metacarpals and metatarsals and distal radius and ulna. The amount of sclerosis in the proximal phalanges varies from much to little and is greatest in the metaphyseal regions. The tarsals have some sclerosis greatest in the cuboids. Pathologic fractures are shown through sclerotic bone in the distal radius (*arrow*) and ulna in the anteroposterior projection (*D*) at 30 months and near nonsclerotic bone in the right femur (*arrows*) at 47 months (*E*).

### Axial skeleton

#### Skull

The neurocranium at ages 22 and 33 months was large, resulting in craniofacial disproportion (Fig. 2). The frontal bone bulged anteriorly; the midface was small. The posterior fossa appeared prominent. Sutures were normal. Closure of the anterior fontanelle seemed delayed (open at 22 months; virtually closed at 33 months). The ascending ramus of the mandible was smaller than expected, and the mandibular angle was obtuse. Some teeth were malformed or abnormally oriented. The skull base was sclerotic, increasing between 7 to 33 months. Periorbital sclerosis was especially prominent along with sclerosis of the greater and lesser wings of the sphenoid and the posterior portion of the sphenoid body. Maxillary and ethmoid sinuses were opaque at ages 22 and 33 months, and the mastoid air cells were small.

#### Head CT

At age 5 months, CT showed a sclerotic skull base but no hydrocephalus or intracranial calcifications. The sulci were slightly enlarged. There was a suggestion of abnormal myelination. The optic foramina, middle ears, and internal auditory canals appeared grossly normal, but detailed optic foramina and petrous bone imaging was not obtained. Multiple follow-up CTs elsewhere were essentially unchanged.

#### Chest

Chest shape was normal, but the ribs were broad and diffusely sclerotic with multiple lower rib fracture deformities at age 7 months. By age 22 months, the ribs were more sclerotic, the medullary cavities were delayed (because they were just beginning to form), and there were multiple healed fractures that had become more numerous in the fifth through the eleventh ribs bilaterally. The ribs were shortened with a modeling error anteriorly, and wide anteriorly and laterally (Fig. 3*A*). Fractures had occurred anteriorly, and laterally and were not remodeling. At 33 months, the ribs were still sclerotic and widened, and they had more recent fractures and multiple fracture deformities but were showing further medullary cavity development. The clavicles were sclerotic, and their medial ends widened at ages 22 and 33 months. The sternal segments were broad and sclerotic, with a central lucency, as were the scapulae. The heart and lungs appeared normal.

#### Pelvis

Sclerosis was accompanied by a normal-appearing central portion of the iliac bones causing a “bone in bone” appearance at age 22 months (Fig. 3*A*), and even more apparent at 33 months. The upper iliac borders were irregular. The ischial and pubic bones were sclerotic. The femoral heads and metaphyses were sclerotic, and the necks were wide with failure of modeling.

#### Spine

The vertebral bodies were sclerotic, with the cranial and caudal ends appearing “sandwich-like,” and their anterior margins were pointed anteriorly. Compared with age 7 months, when the lumbar vertebrae had a more oval shape (Fig. 3*B*), vertebral body flattening was more striking at age 22 months, and at 33 months the vertebral bodies showed rectangular shapes with greater relative flattening (Fig. 3*C*). There was some anterior vertebral notching. The disk spaces were relatively widened. The neural arches also were sclerotic. A pars defect, in keeping with an OPT,(24) was observed at L_4_, but healed by 33 months.

### Appendicular skeleton

#### Extremities

The overall pattern was one of sclerosis focused primarily on the metaphyses that also were expanded (Fig. 4).

Tubulation failure was best seen in the distal femurs (Fig. 4*A, B*). By 33 months, this expansion extended almost to the midshafts. The expansion, which was greatest in the metaphyses, extended into the diaphyses and from sclerotic bone into nonsclerotic bone. Between the ages of 7 and 43 months, the dense sclerosis at the ends of the bones increased appreciably in length. Some areas of metaphyseal sclerosis were sharply demarcated at the diaphysis, whereas others appeared to fade into more normal-appearing bone. There was a healing fracture in the distal left femur at the junction of the sclerotic and nonsclerotic bone, shown by periosteal new bone formation and focal expansion of the bone (Fig. 4*A*). The rate at which extremity fractures healed was delayed. Fractures continued to occur. Transverse lucent areas that were small and faint were seen in the dense sclerosis of some metaphyses, such as the distal femur (Fig. 4*E*).

In the proximal end of the humeral metaphyses and both distal radii and ulnas, between 7 and 33 months of age, there were well-demarcated areas of dense sclerosis that increased in length (Fig. 4*C, D*). The junctions between the sclerotic and nonsclerotic bone were varied, being transverse, oblique, and rounded. The cortices in the midshafts were thickened. The proximal humeri and the distal radii and ulnas showed best the irregularity of the provisional zones of calcification at the physeal plates (Fig. 4*C, D*). The epiphyses were sclerotic.

#### Hands and feet

Sclerosis was greatest in the metacarpals, metatarsals, and distal radii and ulnas and often most in their metaphyses (Fig. 5). Nevertheless, metacarpals and metatarsals contained nonsclerotic areas. Some phalanges were almost entirely sclerotic, whereas others appeared normal. In some, metaphyseal sclerosis terminated abruptly into more normal-appearing bone, and then into a mixed sclerosis pattern. The cuboids had focal sclerosis, as did some other tarsal bones, but the carpals and distal radial epiphyses had no sclerosis. The distal radii and ulnas showed irregular physeal plates, provisional zones of calcification, and broad undertubulated, densely sclerotic metaphyses where the sclerosis terminated abruptly into more normal-appearing bone (Fig. 5*D*). In addition, the sclerosis ended in a rounded or oblique shape. The second metacarpals had accessory epiphyses. The bone age at 22 months was delayed to 18 months. At 33 months, the metaphyses of the phalanges were more sclerotic, as were the epiphyses.

At age 3 years, DXA L_1_–L_4_ spine BMD *Z*-score was +8.1.

#### Fractures

Fractures would occur through radiographically dense bone (Fig. 5*D*) or at the junction of osteosclerosis and less remarkable bone (Fig. 5*E*) and were slow to heal.

### Histopathologic findings

**Fig. 6:**
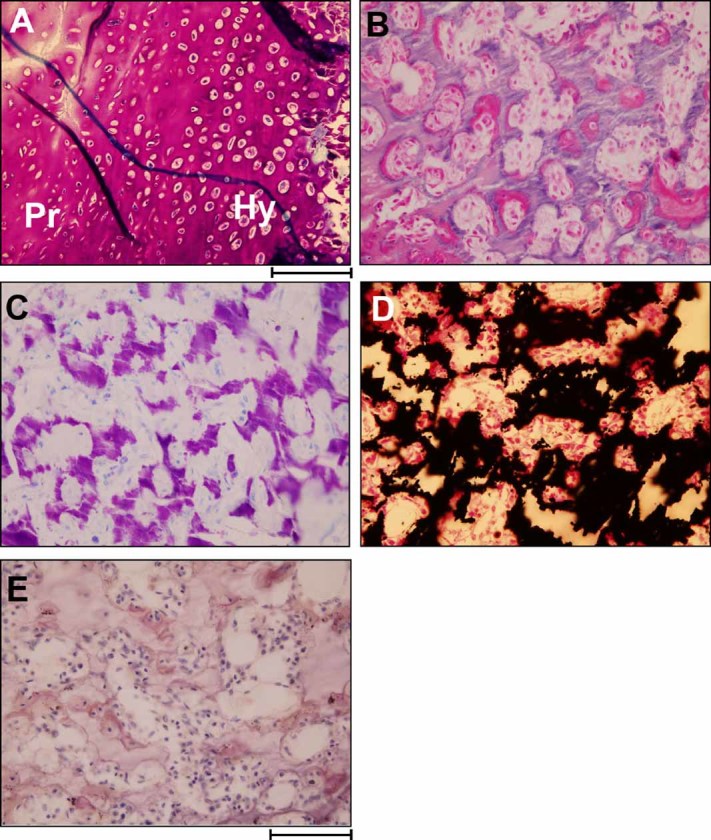
Osteoclast-poor osteopetrosis. Sections of the nondecalcified transapophyseal iliac crest biopsy at age 25 months show a growth plate (*A*) containing proliferative (Pr) and hypertrophic (Hy) chondrocytes that lack the normal columnar orientation along the axis perpendicular to the bone surface. In this toluidine-stained section, the bone surface is toward the left, and the chondrocytes should be arranged in rows from left to right. Scale bar = 200 µm. H&E-stained (*B*) and toluidine blue–stained (*C*) sections demonstrate disorganized trabeculae composed primarily of cartilage (*purple* in both stains), with only minimal bone matrix (*pink* in *B*, *pale gray* in *C*). A von Kossa stain (D) shows that the cartilage in these “trabecular” areas is calcified. TRAP staining (*E*) fails to identify any osteoclasts. Scale bar (*B–E*) = 100 µm.

Transapophyseal iliac crest biopsy showed a disordered growth plate consistent with the findings in some appendicular radiographs (see above). Specifically, the physis had zones of chondrocytes with normal proliferative and hypertrophic morphologies (Fig. 6*A*), but the cells were not arranged in their usual columnar orientation. An area deep to the growth plate, which at the patient's age is normally composed of trabecular bone with modest amounts of centrally located cartilage remnants, consisted almost entirely of calcified cartilage, as shown by H&E, toluidine blue, and von Kossa stains (Fig. 6,*B*–*D*). There was replacement of trabecular bone by calcified cartilage and diminished numbers of hematopoietic cells. Fluorescence imaging of unstained sections (not shown) demonstrated diffuse uptake of demeclocycline in areas of calcified cartilage near the growth plate, but there were no linear single or double labels to indicate normal bone formation. The area between the calcified cartilage contained primarily mesenchymal cells (ie, osteoblasts and fibroblasts), with only rare hematopoietic cells. Osteoclasts were not identified morphologically or by TRAP staining (Fig. 6*E*). Overall, the findings of increased amounts of cartilage, decreased hematopoietic marrow, and an absence of identifiable osteoclasts were consistent with a form of “osteoclast-poor” OPT.

### Family studies

Skeletal survey of the parents revealed no significant abnormalities. The mother, 5 feet, 1 inch tall, had brachycephaly and minimal degenerative changes of L_5_. The father, a 5 foot, 11 inch postman, had brachycephaly, spondylolysis of L_5_, anterior wedging of T_12_, and multiple Schmorl's nodes in his lumbar and thoracic spine. The patient's two brothers and three paternal cousins had several radiographs taken elsewhere following trauma that were essentially unremarkable.

DXA of the mother showed BMD *Z*-scores at the upper half of the age-matched normal range for the L_1_–L_4_ spine, total hip, wrist, and whole body. The father's values were average for gender and age.

Studies of mineral homeostasis were unremarkable for the mother and father. Respectively, serum TRACP-5b was 3 and 2 U/L (normal 2 to 4 U/L for females and 3 to 5 U/L for males), osteocalcin 8 and 5 ng/mL (normal 3 to 14 ng/mL), and BAP 16 and 15 U/L (normal 12 to 31 U/L for females and 15 to 41 U/L for males). Urine free DPD was 5.0 and 2.9 nmol DPD/mmol of Cr (normal 3.0 to 7.4 nmol/mmol for females and 2.3 to 5.4 nmol/mmol for males). The father's profile suggested low-normal remodeling.

### Mutation analyses

Mutation analysis of the patient's *CLCN7*, *TCIRG1*, and *OSTM* genes was notable only for a previously unreported heterozygous missense variation in *CLCN7* (p.T198M). This seemed noncontributory to the DSS because it occurred in the healthy father and would affect an amino acid not conserved across species.(25)

No mutations were detected in *TNFRSF11A*, *TNFRSF11B*, *CSF1*, *CSF1R*, or *ANKH.* However, in *TNFSF11*, encoding RANKL, we found a heterozygous single-base alteration predicting an amino acid change (c.107C > G, p.Pro36Arg). This missense variation also was identified in the patient's father, but not in her mother, and was not listed in dbSNP,(26) nor had it been reported in several association studies of RANKL polymorphisms.(27,28) However, we did find it (heterozygous) in 4 of 67 patients [4 of 134 alleles (3.0%)] with a variety of metabolic/dysplastic bone diseases (see “Discussion”) and homozygous in a man with an unusual, severe variant of Camurati-Engelmann disease.(29) Nevertheless, using this candidate gene approach, we did not identify a causal gene defect for the DSS that we presume was inherited in this family as an autosomal recessive trait. Microarray-based genomic copy-number analysis of the patient and parents also was unrevealing.

### Treatment

Lowering excessive dietary calcium was advised to ensure that circulating PTH levels were not suppressed and thereby impairing bone resorption. Following her second inpatient evaluation, reduction from approximately 950 to 500 mg calcium (RDA) was recommended, but a food record suggested intake of 775 mg. Thereafter, even small changes seemed to explain wide swings in serum PTH levels (Table 1), in keeping with severe OPT.(30)

No recommendation was made for bone marrow transplantation (see “Discussion”). Teriparatide was not prescribed, and PTH seemed ineffective in a previous patient.(6) Particular attention was paid to possible surgical intervention if narrowed cranial foramina caused symptoms.

### Literature review

To date, 23 patients are said to have DSS. In 2008, Lemire and Wiebe(16) found that 17 patients had been reported with DSS(4,6,8–11,15,16,31–35) after Spranger and colleagues(2) had, 40 years earlier, described their one case, four previously reported patients from Ellis in 1934 (case 2),(12) later studied by Field in 1938,(13) and one known to Maroteaux and to Stehr in 1942 (case 2).(36) However, our review showed that the precise diagnoses of the unusual patients of Chitayat and colleagues(37) and Maheshwari and colleagues(38) are somewhat unclear. The patient of Maheshwari and colleagues(38) seems to have a type of craniometaphyseal dysplasia. Undoubtedly, there are more unreported and unrecognized patients. Of historical note, DSS has previously affected a nonconsanguineous Turkish infant girl.(14)

## Discussion

Comprehensive investigation of our patient revealed that DSS presents in infancy as a distinctive “osteoclast-poor” form of OPT associated with reduced bone remodeling.

### History of DSS

DSS was delineated in the German medical literature in 1968 when Spranger and colleagues(2) described an affected 12-year-old boy and reviewed five similar pediatric cases.(12,13,36,39) The principal radiographic features were osteosclerotic and dysplastic abnormalities in long bones similar to Pyle disease (OMIM % 265900)(1) but with characteristic platyspondyly and inconsistent hypoplasia of the iliac bones, also with short stature, bone fragility, dysodonty, and cranial nerve compression.(2) By 2008, there were 23 reported cases.(4,6,8–11,15,16,31–35) Our literature review indicated that nearly all represent DSS.

### Clinical features of DSS

DSS presents in infancy and is readily distinguished from other sclerosing bone disorders by its remarkable early acquired sclerotic platyspondyly, metaphyseal expansion, and demarcated osteosclerosis.(6) Follow-up of one patient(16) to age 44 years revealed that the widened and osteosclerotic metaphyseal areas of the long bones seem, unlike most types of OPT,(3) to eventually become radiolucent. The patient's skull, facial bones, ribs, and spine remain dense, with persisting platyspondyly and vertebral endplate sclerosis.(16)

DSS features bowing of long bones, absence of marrow failure or acroosteolysis,(38) perhaps skin changes (red-violet spots in a patchy distribution),(11–14) developmental regression, and a variable prognosis. There are reports of dental and enamel hypoplasia with delayed or absent tooth eruption and premature loss of teeth.(35) The enamel can be irregular and, of interest, hypercalcified.(35) The dental manifestations of DSS were reviewed by Utz in 1970,(40) and osteomyelitis of the mandible by Packota and colleagues in 1991.(7)

### Radiographic features of DSS

Major radiographic abnormalities in DSS that change with age include thickening and sclerosis of the calvarium and base of the skull, decreased pneumatization of paranasal sinuses and mastoids, and sclerosis of the scapula, clavicles, and ribs that are also broad and contain multiple fracture deformities. The pelvic bones are sclerotic, with the iliac bones having a nonsclerotic center or the “bone-in-bone” appearance. The vertebrae are sclerotic and flattened with anterior body wedging. Preadolescent long bone findings include dense sclerosis of epiphyses, metaphyses that are expanded and club-shaped, but midshafts that are relatively spared. There is irregularity of the physeal plates and the adjacent provisional zones of calcification. In childhood, the hand and foot bones, including the carpals and tarsals, are sclerotic, with the increased density appearing greatest at the ends of the tubular bones. The dense, expanded, Erlenmeyer flask–shaped bone ends evolve into osteopenic widened bones that include portions of the diaphysis with thin cortices. Sclerosis remains focal (largely in the metaphyses) in the long bones, but is present in much of the rest of the skeleton.(6,8,16)

### Differential diagnosis of DSS

Serial radiographs during infancy should distinguish DSS from other conditions, including various OPTs, pyknodysostosis, Camurati-Engelmann disease, and craniometaphyseal dysplasia.(41) The especially helpful (diagnostic) feature in DSS is the flattening of vertebrae with punctate densities. Alternatively, mutation analysis is available for these other disorders.

For DSS, a severe type of OPT is the principal diagnostic consideration.(3) The key features for diagnosis are the spinal osteosclerosis, flattening, and anterior beaking early on, as well as the sharply marginated sclerotic and expanded metaphyses. However, in older (eg, adolescent) DSS patients, osteosclerosis is replaced by radiolucency in the expanded ends of the tubular bones.(6,16) Sclerosis then may involve the diaphyses and appear only focally in the metaphyses.

Eventually, DSS probably will be diagnosed by mutation analysis. DSS is considered to be an autosomal recessive trait (OMIM % 224300).(1,6) However, X-linked recessive inheritance seems possible,(11) although affected girls,(10,31) such as our patient, challenge exclusivity for this pattern.

### Dysosteosclerosis as a form of osteopetrosis

DSS is not classified among the OPTs by the International Skeletal Dysplasia Society.(42) Yet osteoclast failure is unequivocal in DSS, with persistence of unresorbed primary spongiosa (calcified cartilage bars or islands) revealed by iliac crest biopsy. How this finding occurs with increasing osteosclerosis in most, but not all, areas of the skeleton remains an enigma. DSS affects especially endochondral bone formation, but also intramembranous bone formation(33) and perhaps development of the teeth.(4) Our patient's sequential radiographs indicated that failed osteoclast activity began abruptly and then could be profound. Our patient had bone-within-bone appearances on radiographs (especially the iliac bones), representing another feature of OPT. Faint transverse banding in several metaphyseal areas suggested slight fluctuation of compromised bone remodeling, as can be seen in Albers-Schönberg disease (ie, “benign” OPT).(43) Our patient's recurrent fractures through dense bones, as in other OPTs, probably resulted from retention of cartilage rests, failure of osteons to interconnect during remodeling, and compromised microfracture repair. Our patient also had a pars defect in keeping with the brittleness of OPT.(24) Furthermore, in DSS, bone histology has shown a paucity of both osteoclasts and osteoblasts(11) and, after tetracycline double labeling, a mineralization abnormality featuring large amounts of osteoid with diffuse tetracycline labels.(4)

Failure of osteoclast-mediated external shaping of long bones (modeling or tubulation) in any OPT leads to expanded metaphyses with an Erlenmeyer flask deformity.(44) Marrow cavity compromise occurs because of failure of endosteal bone resorption,(45) but to date we have not found dysfunction of hematopoiesis in our patient. Facial sinuses do not form properly and aerate, likely explaining her nasal “stuffiness.” Similarly, neural foramina in the skull do not widen in OPT, but our patient has not manifested cranial nerve compression.

In our patient, in keeping with her relatively young age of 3¾ years, sequential radiographs revealed the increasing metaphyseal expansion and osteosclerosis of DSS. Her other major radiographic changes were sclerosis of the base of her skull and around the orbits, vertebral body sclerosis with worsening flattening and anterior pointing, sclerosis of the ribs with loss of medullary space and multiple healed fractures, long bone metaphyseal sclerosis, expansion, and failure of modeling. These findings indicated acquired, early postnatal osteoclast failure. At age 7 months, our patient's radiographs showed that “older” skeletal regions contained relatively normal bone. Subsequently, remarkable demarcation occurred as osteoclast activity failed. Notably, serum BAP, osteocalcin, and TRACP-5b all were low, consistent with slow bone remodeling as osteoclast activity decreased. Absence of osteoclasts would preclude their signaling to osteoblasts. Our biochemical findings are in keeping with a postnatal OPT, especially one featuring few osteoclasts, with low or low-normal serum levels of markers of bone turnover.(46) Consonant with reports of DSS, our patient's markers of bone remodeling matched her radiographic and histopathologic findings and indicated suppression of skeletal turnover, including bone formation. Histopathologic study revealed an associated defect in mineralization.

Notably, her serum CK-BB was normal, and TRACP-5b was persistently low in contrast to other forms of OPT(17) featuring defects intrinsic to osteoclasts, where CK-BB and TRACP5b are elevated. Furthermore, unlike in Albers-Schönberg disease, due to inactivating mutations in *CLCN7*, serum LDH and AST levels were normal (not elevated).(17)

In DSS, remarkable resolution of appendicular osteosclerosis over time might question classification as an OPT. However, in the carbonic anhydrase II (CA II) deficiency form of OPT in which osteoclasts do not acidify the pericellular milieu, this finding does occur,(47) perhaps from the chronic metabolic acidosis. Our patient did not manifest the renal tubular acidosis that characterizes CA II deficiency, and therefore CA II levels in erythrocytes or mutation analysis of *CA II* were not performed. The acquired reversal of osteoclast failure in DSS remains unexplained.

### Genetic basis of DSS

Although DSS is extremely rare, autosomal recessive inheritance seems certain for at least some cases because of reports of consanguinity.(15) Our patient's family history was negative for consanguinity, which was absent for another Turkish infant girl with DSS.(10,14)

No mutations were found in our patient in *CLCN7* or *TCIRG1*, that account for most human OPT,(48) or in *OSTM1*. Unlike most human forms of OPT, where dysfunctional osteoclasts are present or abundant,(46,47) deficiency of osteoclasts appears to account for OPT in our DSS patient. Osteoclast precursor cells seem to be available because our patient's circulating monocyte counts were repeatedly normal, but we did not test osteoclastogenesis from CD14 cells. Insufficiency of osteoclasts causing OPT has been observed in humans only for RANKL(21) and RANK(48) deficiency (see below). This finding has crucial therapeutic implications for DSS because bone marrow transplantation (BMT) might not help.(46) Accordingly, we sequenced the exons and splice junctions of *TNFRSF11A* (RANK), *TNFRSF11B* (OPG), and *TNFSF11* (RANKL) and found no mutations.

Because the OPT from an insertion mutation within *m-csf* in the op mouse resolves over time,(49) we studied our patient's *CSF1* gene encoding M-CSF and the *CSF1R* gene encoding its receptor C-FMS but found no abnormality. Because DSS seems to share some of the radiographic features of craniometaphyseal dysplasia by the teenage years, including metaphyseal widening and craniofacial sclerosis,(41) we studied *ANKH*, but the findings were unremarkable.

Bone-formation rates are reduced in osteoclast-poor forms of OPT, according to biochemical markers and histopathology.(50) In fact, markers in our patient were repeatedly remarkable for very low serum osteocalcin and TRACP-5b levels. However, the high-bone-density phenotype of the osteocalcin knockout mouse does not appear to be an OPT.(51) Hence we did not sequence our patient's osteocalcin gene.

RANK gene (*TNFRSF11A*) analysis was unrevealing for our patient's osteoclast-poor OPT. Additionally, there was no evidence of a *TNFSF11* splice site or exonic defect involving RANKL but instead a likely rare polymorphism. Our patient carries a heterozygous variation (P36R) in RANKL that represents a nonconservative amino acid change in a conserved region of this molecule.(52) P36 is part of a proline-rich region (7 proline residues/10-amino-acid stretch) near the intracellular side of the transmembrane domain. However, we do not know of any function for this 10-amino-acid proline-rich region in RANKL. This *TNFSF11* change was not found in dbSNP(26) and was not reported in several association studies of RANKL single-nucleotide polymorphisms (SNPs) with bone phenotypes.(27,28,53) However, we identified this RANKL variant in 4 of 67 heterozygous patients (or family member) with various bone diseases and 1 homozygous patient with a unique variant of Camurati-Engelmann disease,(29) but a healthy population has not been screened. It may represent a rare polymorphism conditioning the DSS phenotype of our patient.

Thus, we were unable to discover a genetic basis for DSS using this candidate gene approach in this patient. A considerable number of animal models for OPT lack osteoclasts and thus provide further candidate genes,(46,47) including *TRAF6*, *FOS*, *JUN*, *NFATc1*, *NFκB* subunits, and *RGS1D*.(42,46) Microarray studies were not revealing, but homozygosity mapping may be useful. Gene expression studies using DSS monocytes are planned, which may help to identify the gene defect(s). Negative mutation analyses in our single patient do not exclude involvement of the tested genes in other DSS patients because there may be genetic heterogeneity for this disorder.

### Treatment of DSS

Because our patient has osteoclast deficiency, we did not advise BMT, which has been used for severe OPT when the pathogenic defect is intrinsic to these cells.(45,48) In RANKL-deficiency OPT, where few osteoclasts form, BMT has not been helpful.(21) In fact, one DSS patient died at age 10 months from complications of BMT.(31) Furthermore, we have not advised teriparatide in part because of our patient's paucity of osteoclasts. The patient of Houston and colleagues(6) did not respond biochemically with hypercalcemia, etc. following intramuscular injections of PTH. Our patient's management has been largely orthopedic, but we recommended control of her excessive dietary calcium to ensure that circulating levels of PTH are not suppressed and thereby blocking bone resorption. Although the few case reports of DSS leave much uncertainty concerning the natural history, bone remodeling may become active later in childhood.

## References

[1] Online Mendelian Inheritance in Man (OMIM) http://www.ncbi.nlm.nih.gov/omim/.

[2] Spranger J, Albrecht C, Rohwedder HJ, Wiedemann HR (1968). Dysosteosclerosis: a special form of generalized osteosclerosis. Fortschr Geb Rontgenstr Nuklearmed..

[3] Whyte MP, Royce PM, Steinmann B Osteopetrosis. Connective Tissue and Its Heritable Disorders: Molecular, Genetic, and Medical Aspects.

[4] Leisti J, Kaitila I, Lachman RS, Asch MJ, Rimoin DL (1975). Dysosteosclerosis. Birth Defects..

[5] Kaitila I, Rimoin DL (1976). Histologic heterogeneity in the hyperostotic bone dysplasias. Birth Defects..

[6] Houston CS, Gerrard JW, Ives EJ (1978). Dysosteosclerosis. Am J Roentgen..

[7] Packota GV, Shiffman J, Hall JM (1991). Osteomyelitis of the mandible in a patient with dysosteosclerosis. Oral Surg, Oral Med, Oral Path..

[8] Lachman RS Dysosteosclerosis. Taybi and Lachman's: Radiology of Syndromes, Metabolic Disorders, and Skeletal Dysplasias.

[9] Spranger JW, Brill PW, Poznanski A Dysosteosclerosis. Bone Dysplasias: An Atlas of Genetic Disorders of Skeletal Development.

[10] Sener RN, Yalman O, Cetingul N, Tutuncuoglu S, Kavakli K, Ustun EE (1997). Dysosteosclerosis: clinicoradiologic findings including brain MRI. Comput Med Imaging Graph..

[11] Pascual-Castroviejo I, Casas-Fernandez C, Lopez-Martin V, Martinez-Bermejo A (1997). X-linked dysosteosclerosis. Four familial cases. Eur J Pediatr..

[12] Ellis RWB (1934). Osteopetrosis. Proc Roy Soc Med..

[13] Field CE (1938). Albers-Schönberg Disease. An atypical case. Proc Roy Soc Med..

[14] Roy C, Maroeaux P, Kremp L, Courtecuisse V, Alagille D (1968). Un nouveau syndrome osseux avec anomalies cutanées et troubles neurologiques. Arch Fr Pediatr..

[15] Johns E, Kozlowski K, Masel J, Muralinath S, Vijayalaskshmi G (1998). Dysosteosclerosis. Australas Radiol..

[16] Lemire EG, Wiebe W (2007). Clinical and radiologic findings in an adult male with dysosteoslcerosis. Am J Med Genet..

[17] Whyte MP, Kempa L, McAlister WH, Zhang F, Mumm S, Wenkert D (2010). Elevated serum lactate dehydrogenase isoenzymes and aspartate transaminase distinguish Albers-Schönberg disease (chloride channel 7 deficiency osteopetrosis) among the sclerosing bone disorders. J Bone Miner Res.

[18] Whyte MP, Chines A, Silva DP, Landt Y, Ladenson JH (1996). Creatine kinase brain isoenzyme (BB-CK) presence in serum distinguishes osteopetroses among the sclerosing bone disorders. J Bone Miner Res..

[19] Hughes AE, Ralston SH, Marken J (2000). Mutations in TNFRSF11A, affecting the signal peptide of RANK, cause familial expansile osteolysis. Nat Genet..

[20] Whyte MP, O'Brecht SE, Finnegan PM (2002). Osteoprotegerin deficiency and juvenile Paget's disease. N Engl J Med..

[21] Sobacchi C, Frattini A, Guerrini MM (2007). Osteoclast-poor human osteopetrosis due to mutations in the gene encoding RANKL. Nature Genet..

[22] Nürnberg P, Thiele H, Chandler D (2001). Heterozygous mutations in ANKH, the human ortholog of the mouse progressive ankylosis gene, result in craniometaphyseal dysplasia. Nat Genet..

[23] Turan S, Bereket A, Omar A, Berber M, Ozen A, Bekiroglu N (2005). Upper segment/lower segment ratio and armspan-height difference in healthy Turkish children. Acta Paediatr..

[24] Martin RP, Deane RH, Collett V (1997). Spondylolysis in children who have osteopetrosis. J Bone Joint Surg Am..

[26] http://www.ncbi.nlm.nih.gov/sites/entrez?db=snp.

[27] Xiong DH, Shen H, Zhao LJ (2006). Robust and comprehensive analysis of 20 osteoporosis candidate genes by very high-density single-nucleotide polymorphism screen among 405 white nuclear families identified significant association and gene-gene interaction. J Bone Miner Res..

[28] Hsu YH, Niu T, Terwedow HA (2006). Variation in genes involved in the RANKL/RANK/OPG bone remodeling pathway are associated with bone mineral density at different skeletal sites in men. Hum Genet..

[29] Whyte MP, Totty WG, Novack DV, Wenkert D, Zhang X, Mumm S Camurati-Engelmann disease: unique variant featuring a novel mutation in TGFß1 encoding transforming growth factor beta 1 with a missense change in TNFSF11 encoding RANK ligand. J Bone Miner Res..

[30] Key L, Carnes D, Cole S (1984). Treatment of congenital osteopetrosis with high-dose calcitriol. N Engl J Med..

[31] Elçioglu NH, Vellodi A, Hall CM (2002). Dysosteosclerosis: A report of three new cases and evaluation of the radiological findings. J Med Genet..

[32] Fryns JP, Vinken L, Claessens S, Marien J, Geutiens J, Van den Berghe H (1980). Dysosteosclerosis in a mentally retarded boy. Acta Paediatr Belg..

[33] Greenspan A (1991). Sclerosing bone dysplasias–a target-site approach. Skeletal Radiol..

[34] Canepa G, Maroteaux P, Pietrogrande V (2001). Dysmorphic Syndromes and Constitutional Diseases of the Skeleton.

[35] Oncag O, Ozkinay FF, Eronat C (1999). A case with unique dental findings and SEM evaluation of a hypoplastic tooth. J Clin Pediatr Dent..

[36] Stehr L (1942). Pathogenese und Klassifikation der Osteoskleresen. Arch Orthop Unfall-Chir..

[37] Chitavat D, Silver K, Azous EM (1992). Skeletal dysplasia, intracerebral calcifications, optic atrophy, hearing impairment, and mental retardation: nosology of dysosteosclerosis. Am J Med Genet..

[38] Maheshwari A, Rao KK, Kohli N (1996). Case report: dysosteosclerosis: a unique entity. Clin Radiol..

[39] Alagille D

[40] Utz VW (1970). Manifesetation der Dysosteosklerose in Kieferbe-reich. Dtsch Zahnaerztl Z..

[41] Bartuseviciene A, Samuilis A, Skucas J (2009). Camurati-Engelmann disease: imaging, clinical features and differential diagnosis. Skeletal Radiol..

[42] Superti-Furga A, Unger S, and the Nosology Group of the International Skeletal Dysplasia Society (2007). Nosology and classification of genetic skeletal disorders: 2006 version. Am J Med Genet Part A..

[43] Whyte MP, Rosen CJ, Compston JE, Lian JB (2008). Primer on the Metabolic Bone Diseases and Disorders of Mineral Metabolism.

[44] Faden MA, Krakow D, Ezgu F, Rimoin DL, Lachman RS (2009). The Erlenmeyer flask bone deformity in the skeletal dysplasias. Am J Med Genet..

[45] Tolar J, Teitelbaum SL, Orchard PJ (2004). Osteopetrosis. N Engl J Med..

[46] Villa A, Guerrini MM, Cassani B, Pangrazio A, Sobacchi C (2009). Infantile malignant, autosomal recessive osteopetrosis: the rich and the poor. Calcif Tissue Int..

[47] Whyte MP, Murphy WA, Fallon MD (1980). Osteopetrosis, renal tubular acidosis, and basal ganglia calcification in three sisters. Am J Med..

[48] Guerrini MM, Sobacchi C, Cassani B (2008). Human osteoclast-poor osteopetrosis with hypogammaglobulinemia due to TNFRSF11A (RANK) mutations. Am J Hum Genet..

[49] Yoshida H, Hayashi S, Kunisada T (1990). The murine mutation osteopetrosis is in the coding region of the macrophage colony stimulating factor gene. Nature..

[50] Segovia-Silvestre T, Neutzsky-Wulff AV, Sorensen MG (2009). Advances in osteoclast biology resulting from the study of osteopetrotic mutations. Hum Genet..

[51] Boskey AL, Gadaleta S, Gundberg C, Doty SB, Ducy P, Karsenty G (1998). Fourier transform infrared microspectroscopic analysis of bones of osteocalcin-deficient mice provides insight into the function of osteocalcin. Bone..

[52] Venot C, Maratrat M, Dureuil C, Conseiller E, Bracco L, Debussche L (1998). The requirement for the p53 proline-rich functional domain for mediation of apoptosis is correlated with specific PIG3 gene transactivation and with transcriptional repression. EMBO J..

[53] Soedarsono N, Rabello D (2006). Evaluation of RANK/RANKL/OPG gene polymorphisms in aggressive periodontitis. J Periodontal Res..

